# Plasma biomarker analysis of ovarian cancer patients undergoing immunotherapy with therapeutic cancer vaccine DPX-Survivac

**DOI:** 10.1186/2051-1426-3-S2-P95

**Published:** 2015-11-04

**Authors:** Mohan Karkada, Devanand Pinto, Eve Teh, Kenneth Chisholm, Tara Quinton, Neil Berinstein, Marc Mansour

**Affiliations:** 1 Immunovaccine Inc., Halifax, NS, Canada; 2 National Research Council - Human Health Therapeutics, Halifax, NS, Canada; 3 Sunnybrook Health Sciences Centre, Toronto, ON, Canada

## Background

Immune based therapies for cancer are emerging as promising strategies to treat a number of malignancies. A more thorough biomarker assessment of the patients may provide insights into the variability in response to such immune therapies. In a Phase I/Ib clinical trial with the survivin-targeting vaccine DPX-Survivac, we showed the induction of robust immune responses in ovarian cancer patients. In this study, we investigated the potential of proteomic and metabolomic analysis of plasma to identify surrogate biomarkers for efficacy, recurrence and adverse events (AE) in vaccine recipients.

## Methods

Plasma from patients enrolled in a Phase I (n=18) or Phase Ib (n= 22) trial was collected at baseline and post-treatment time points. Metabolomic analysis was conducted using a targeted assay for 58 metabolites by liquid chromatography coupled to tandem mass spectrometry (LC-MS/MS). Proteomic analysis was performed by depletion of albumin and IgG, protein digestion with trypsin, labeling with stable isotopes (TMT reagents) followed by quantitative proteomic analysis by LC-MS/MS. Data analysis was conducted using Skyline, Bioconductor suite and in-house R algorithms in order to correlate profiles with treatment outcomes.

## Results

Plasma metabolomic profiles correlated with the vaccine dosing and the treatment schedule. Of the 58 metabolites that passed the quality control threshold, a panel of four metabolites was found to correlate with recurrence whereas a panel of five metabolites correlated with Grade 3 injection site reaction (ISR). The proteomic analysis resulted in the identification and quantification of 158 plasma proteins. Four proteins correlated with recurrence while another four proteins correlated with ISR. Notably, the levels of proteins and metabolites at the study baseline samples were most indicative of recurrence and ISR.

## Conclusions

The use of metabolomic and proteomic techniques can complement traditional methods for immune monitoring in clinical trials for cancer vaccines and other immune therapies. We demonstrate that a panel of metabolites and proteins in plasma measured at the study baseline correlate with the potential for recurrence and AE. Potential association of novel biomarkers with the strength of immune response could be used for developing an informed approach in patient selection and to personalize treatment options. Due to relatively smaller samples size in this study, these findings require validation in a larger patient cohort.

